# Supplementary material for: Increased Presence of Complement Factors and Mast Cells in Alveolar Bone and Tooth Resorption
Source: Int J Mol Sci. 2021 Mar 9;22(5):2759. doi: 10.3390/ijms22052759 (PMC7967164; doi:10.3390/ijms22052759)
Supplement: Supplementary file 1 [file ijms-22-02759-s001.pdf]

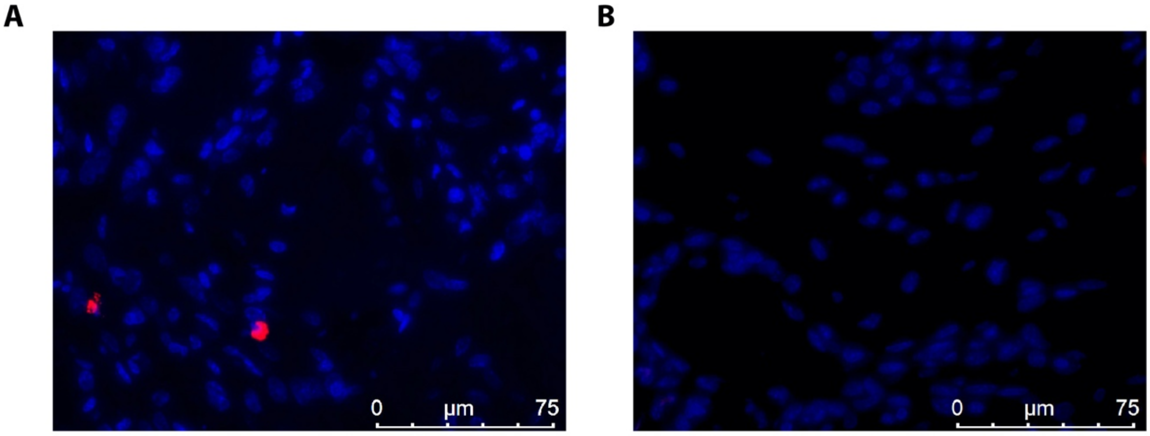

Supplemental Figure S1. Establishment of specific mast cell staining by Avidin-Texas Red conjugate. (**A**) Femur section from a wildtype mast cell competent mouse. Mast cells in the bone marrow are stained with Avidin-Texas Red conjugate. (**B**) Femur section from a Mcpt5-Cre^{+} R-DTA$^{flox/flox}$ mast cell deficient mouse. No cells are stained by Avidin-Texas Red conjugate.
